# The utility of orbital imaging in the evaluation of orbital disease

**DOI:** 10.1371/journal.pone.0308528

**Published:** 2024-08-30

**Authors:** Min Joung Lee, Rohan Verma, Bronwyn E. Hamilton, David Pettersson, Dongseok Choi, Eun Soo Kim, Bobby S. Korn, Don O. Kikkawa, James T. Rosenbaum

**Affiliations:** 1 Department of Ophthalmology, Hallym University Sacred Heart Hospital, Anyang, Republic of Korea; 2 Casey Eye Institute, Oculofacial Plastic and Reconstructive Surgery, Casey Aesthetic Facial Surgery Center, Oregon Health & Science University, Portland, OR, United States of America; 3 Casey Eye Institute, Oregon Health & Science University, Portland, OR, United States of America; 4 Department of Radiology, Oregon Health & Science University, Portland, OR, United States of America; 5 Oregon Health & Science University–Portland State University School of Public Health, Oregon Health & Science University, Portland, OR, United States of America; 6 Department of Radiology, Hallym University Sacred Heart Hospital, Anyang, Republic of Korea; 7 Department of Ophthalmology, Division of Oculofacial Plastic and Reconstructive Surgery, UC San Diego Shiley Eye Institute, La Jolla, CA, United States of America; 8 Devers Eye Institute, Legacy Health System, Portland, OR, Unites Stated of America; 9 Department of Medicine, Oregon Health & Science University, Portland, OR, United States of America; 10 Corvus Pharmaceuticals, Burlingame, CA, United States of America; King’s College Hospital NHS Trust: King’s College Hospital NHS Foundation Trust, UNITED KINGDOM OF GREAT BRITAIN AND NORTHERN IRELAND

## Abstract

**Purpose:**

This study investigates the accuracy of either computerized tomography (CT) or magnetic resonance imaging (MRI) for the evaluation of various orbital diseases.

**Methods:**

We collected 126 CT scans and 65 MRI scans from 144 subjects and asked two radiologists to interpret the images without clinical information. Images included 14 with a clinical diagnosis of orbital infection, 144 with orbital inflammation, and 33 with orbital neoplasm. The inflammatory diseases included thyroid eye disease (TED, n = 69), non-specific orbital inflammation (NSOI, n = 44), IgG4-related disease (IgG4-RD, n = 15), sarcoidosis (Sarcoid, n = 9), granulomatosis with polyangiitis (GPA, n = 5), and Erdheim-Chester disease (ECD, n = 2).

**Results:**

The balanced accuracy (BA) for the two radiologists ranged from 0.87 to 0.90 for cellulitis, 0.81 to 0.86 for inflammation, and 0.82 to 0.85 for neoplasm. Radiologists were excellent at recognizing GPA (BA = 0.98 to 0.99) and very good for TED (BA = 0.80 to 0.86). They also did well identifying IgG4-RD (BA = 0.75 to 0.77), but slightly less well for NSOI (BA = 0.69 to 0.75) and poorly for Sarcoid (BA = 0.48 to 0.50).

**Conclusions:**

CT or MRI scanning contributes to the evaluation of patients with orbital disease, but accuracy does varies based depending on the diagnosis. We could not evaluate issues such as determination of disease activity, variability based on the unit used for imaging or the skills beyond those of our two specialized neuroradiologists. Future studies should directly compare the two imaging modalities and assess the utility of imaging to determine disease activity.

## Introduction

An accurate diagnosis of orbital inflammatory disease is sometimes challenging to ophthalmologists. Clinical symptoms and signs of orbital inflammatory diseases often overlap, and even orbital infection and infiltrative neoplasm can be clinically indistinguishable from orbital inflammation [1,2]. The diagnostic process for orbital diseases includes history taking, physical examination, blood tests, imaging studies, and histopathology. Blood tests are useful to diagnose several orbital inflammations associated with systemic diseases including thyroid eye disease (TED) [3,4], IgG4-related disease (IgG4-RD) [5,6], and granulomatosis with polyangiitis (GPA) [7,8]. However, blood tests have limited diagnostic value in most orbital diseases. While histopathology is considered essential for the definitive diagnosis of orbital diseases, orbital biopsy is not recommended to all patients with an orbital lesion due to its potential risks of injury to the optic nerve or extraocular muscles. Specimens acquired from the orbital biopsies are usually very small such that a specific diagnosis such as GPA cannot always be driven by histopathologic examinations [9].

Radiologic imaging is routinely conducted for the evaluation of orbital diseases and provides essential information in the process of diagnostic work up, but the accuracy of imaging alone for diagnosing various orbital diseases has rarely been investigated. Our group previously reported on the value of radiologic diagnosis for the diagnosis of common orbital inflammatory diseases [10]. However, the need to identify infection or neoplasm is clinically more important because the treatment strategies are substantially different among these disease categories.

In this study, we aimed to determine the accuracy of radiologic diagnosis in terms of classifying 3 orbital disease categories: infection, inflammation, and neoplasm. Then, subgroup analysis was conducted focusing on the orbital inflammation category. The accuracy of radiologic diagnosis was calculated for each specific orbital inflammatory disease. In addition, the abilities of computed tomography (CT) and magnetic resonance image (MRI) to differentiate orbital inflammation and orbital neoplasm were separately calculated and compared.

## Materials and methods

We collected CT and MRI orbital scans, demographic and clinical data of patients who were diagnosed with various orbital diseases from 3 tertiary care institutes: Oregon Health & Science University (OHSU) (Portland, OR, USA), University of California San Diego (UCSD) (La Jolla, CA, USA), and Hallym University Medical Center (HUMC) (Anyang, Korea). The data were accessed for research purposes from 23/05/2020 to 17/11/2021 in OHSU, from 30/05/2018 to 15/09/2018 in UCSD, and from 12/02/2019 to 31/12/2020 in HUMC. This study adhered to the tenets of the Declaration of Helsinki and the tenets of the Health Information Portability and Accountability Act. The protocol was approved and the requirement for written informed consent was waived due to the retrospective nature of the research by the Institutional Review Board of each participating institutes: the institutional review board of Oregon Health & Science University (IRB No. OHSU#6301), the institutional review board of University of California San Diego (IRB No. UCSD#171288X), and the institutional review board of Hallym University Medical Center (IRB No. HUMC 2018-12-002).

Two experienced neuro-radiologists (BEH and DP) reviewed and interpreted orbital imaging blinded to all demographic, clinical, or laboratory data. They were told that the set of imaging exams included all causes of orbital diseases. The radiologic diagnosis was made by a two-step approach. First, the neuroradiologists requested to choose one of the 3 orbital disease categories: orbital infection, orbital inflammation, or orbital neoplasm solely based on the radiologic findings. Second, if they judged that the image corresponded to the orbital inflammation category, they ranked the 3 most likely diagnoses. The radiologists made a diagnosis based on characteristic features of the diagnoses on imaging including anatomical locations involved, density or signal characteristics, enhancement pattern, mass effect, and bone changes. Representative radiologic characteristics of specific orbital diseases are summarized in Table 1.

**Table 1:** Radiologic observations supporting the diagnosis of specific orbital diseases.

Orbital disease	Imaging characteristics
TED	Enlargement of the extraocular muscles (compared to normative data) Pattern of extraocular muscle involvement (inferior > medial > superior oblique > lateral rectus muscles & bilaterality) Fatty infiltration of extraocular muscles (low density on CT &/or high signal intensity on T1- and T2-weighted images) Proptosis &/or increased orbital fat
IgG4-RD	Dacryoadenitis (overlapped with sarcoid, Sjogren’s, and lymphoma) • Enlarged enhancing lacrimal glands with preserved internal morphology, mostly bilateral • Isodensity to brain grey matter, homogeneous enhancement pattern on CT • Iso-intense on T1-weighted images and hypo-intense on T2-weighted images on MRI Trigeminal nerve enlargement Sinus inflammatory disease (overlapped with GPA) Other salivary gland (especially parotid) enlargement and/or nodularity (overlapped with sarcoid, Sjogren’s, and lymphoma) Intracranial findings: Dural thickening or enhancement
NSOI	Heterogeneous Diffuse or ill-defined orbital fat infiltration rather than a localized mass Isolated or disproportionate enlargement of lateral rectus (overlapped with IgG4-RD or malignancy) Extraocular muscle involvement with strong enhancement and surrounding infiltration and involvement of tendinous insertions MRI •Hypo to iso-intense on T1-weighted image •Varied signal intensity on T2-weighted image depending on tissue composition • No diffusion restriction on MRI
GPA	Pan-sinus mucosal thickening Paranasal sinus wall bony destruction, often with septal perforation Sinus obliteration with dense bony thickening and sclerosis Variable orbital soft tissue extension, typically bilateral
Orbital infection	Orbital fat stranding Edema in preseptal±postseptal tissue Rim enhancing fluid collection Adjacent sinusitis
Orbital neoplasm	Rounded/nodular or irregular lesion in shape with loss of normal internal architecture CT • Soft tissue density, Solid enhancement. Bony erosion or destruction • Mass effect on adjacent structures (e.g. globe deformity) MRI • Iso-intense-hypo-intense relative to muscle on T1-weighted image • Hyper-intense on T2-weighted image • Diffusion restriction within a solid mass (especially in lymphoma)

TED, Thyroid eye disease; IgG4-RD, IgG4-related disease; NSOI, Non-specific orbital inflammation; GPA, Granulomatosis with polyangiitis.

Clinical diagnosis was the reference standard. All cases with orbital infection were orbital cellulitis related to paranasal sinusitis. The diagnoses in the orbital neoplasm category were made by histopathologic review. Regarding orbital inflammation, clinical diagnosis was made according to the diagnostic criteria relevant to each diagnosis. The diagnostic criteria of TED, NSOI, IgG4-related ophthalmic disease (IgG4-ROD), sarcoidosis (Sarcoid), and GPA were specified in our earlier report [10]. Erdheim-Chester disease (ECD) was diagnosed by characteristic histopathological findings including infiltration by foamy or lipid-laden histiocytes with admixed or surrounding fibrosis.

The balanced accuracy of radiologic assessment was calculated for each orbital disease category. The diagnostic accuracy of CT and MRI were also described. Regarding subgroup analyses of the orbital inflammation category, the first-documented most likely diagnosis was used as a radiologic diagnosis for the calculation of diagnostic accuracy. All calculations were carried out using the R project (http://www.r-project.org).

## Results

We collected 191 imaging exams from 144 patients (118 scans from OHSU, 41 scans from UCSD and 32 scans from HUMC). Imaging modalities included CT (126 scans) and MRI (65 scans). The orbital disease categories and all clinical diagnoses included in this study are shown in Table 2. The orbital disease categories included orbital infection (14 scans, 7.3%), orbital inflammation (144 scans, 75.4%), and orbital neoplasm (33 scans, 17.3%). The orbital inflammation category consisted of TED (69 scans, 47.9%), NSOI (44 scans, 30.6%), IgG4-ROD (15 scans, 10.4%), Sarcoid (9 scans, 6.3%), GPA (5 scans, 3.5%), and ECD (2 scans, 1.4%). The most common diagnosis in orbital neoplasm category was MALT type orbital lymphoma (12 scans, 36.3%), followed by diffuse large B-cell lymphoma (7 scans, 21.2%), metastatic tumor (3 scans, 9.1%), and meningioma (3 scans, 9.1%).

**Table 2:** Three categories of orbital disease and specific diseases included.

Clinical diagnosis category	Number of scans (% of total scans)	Specific diagnoses (number of scans)
Orbital infection	14 (7.3%)	Orbital cellulitis (n = 14)
Orbital inflammation	144 (75.4%)	Thyroid eye disease (n = 69) Non-specific orbital inflammation (n = 44) IgG4-related disease (n = 15) Sarcoidosis (n = 9) Granulomatosis with polyangiitis (n = 5) Erdheim-Chester Disease (n = 2)
Orbital neoplasm	33 (17.3%)	Orbital lymphoma, MALT (n = 12) Orbital lymphoma, DLBL (n = 6) Metastatic tumor (n = 4) Meningioma (n = 3) Atypical lipomatous tumor (n = 2) Primitive Neuro-ectodermal Tumor (n = 2) Orbital lymphoma, lymphoplasmacytic (n = 1) Plasmacytoma (n = 1) Langerhans cell histiocytosis (n = 1) Squamous cell carcinoma (n = 1)

**Fig 1:**
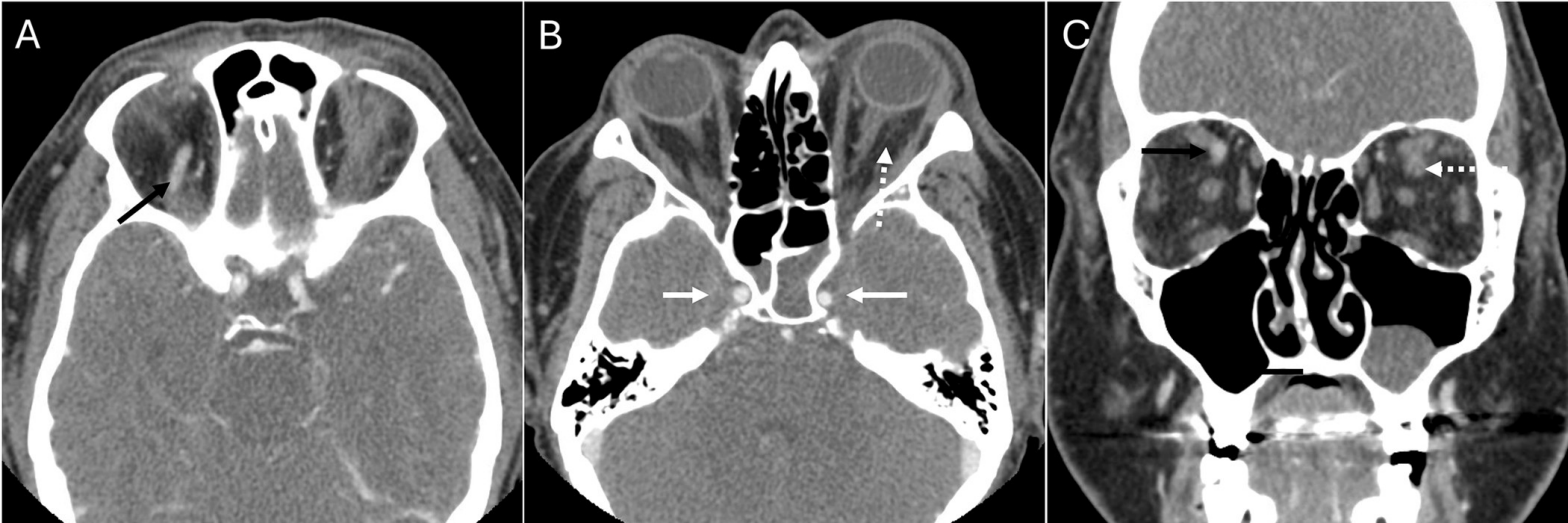
Orbital infection. Axial (A, B) and coronal (C) contrast-enhanced computed tomography scans of a 43 year-old female show left proptosis, post-septal stranding (dashed arrow, B), and a non-enhancing left superior ophthalmic vein (SOV, dashed arrow,C), compared to normally enhanced right SOV (black arrow, A,C). Opacified sphenoid sinuses suggest an infectious source and lack of opacification in the adjacent cavernous sinuses (white arrows, B) raises concern for thrombosis. Both radiologists diagnosed this case as an orbital infection.

**Fig 2:**
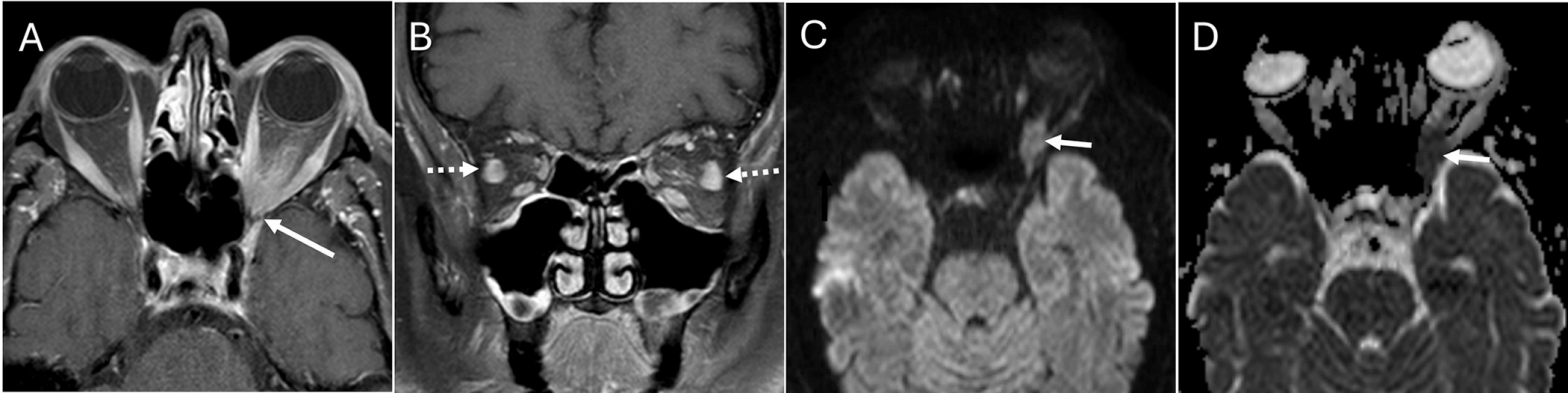
Orbital inflammation. Axial (A) and coronal (B) T1 post-contrast fat saturated images in a 72 year-old male show a left orbital apex mass extending intracranially (white arrows, A,C,D). Mild inflammatory stranding in the pre- & post-septal orbit and mild lateral rectus muscle enlargement (dashed white arrows, B) led one radiologist to conclude that the orbital apex mass was inflammatory, while the other radiologist felt the mass-like appearance favored neoplasm. Diffusion-weighted imaging (DWI) (C) and apparent diffusion coefficient (ADC) (D) show intermediate signal close to brain(white arrows). The confirmed clinical diagnosis was non-specific orbital inflammation.

**Fig 3:**
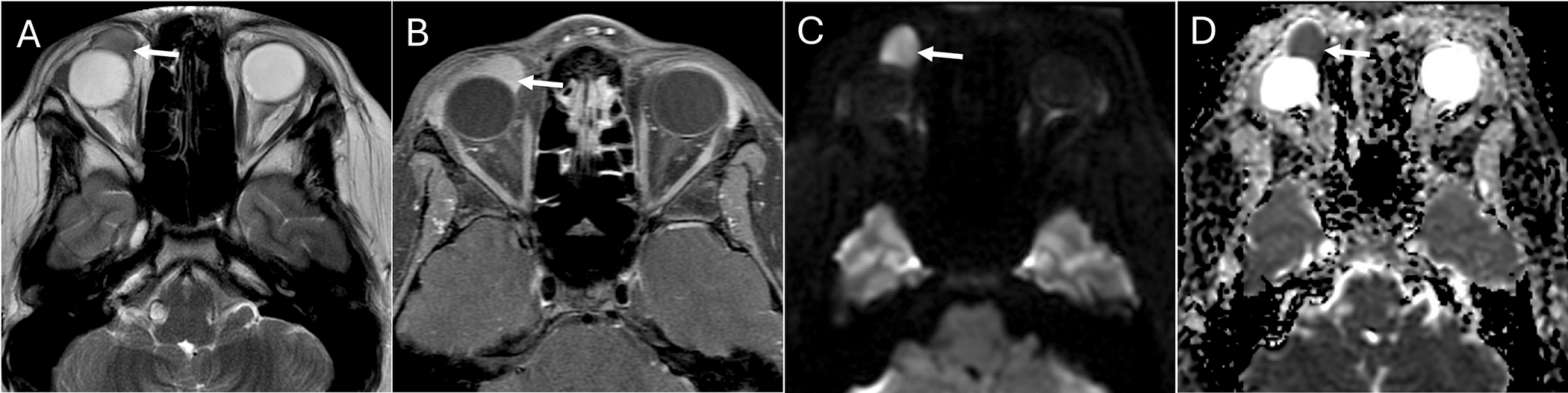
Orbital neoplasm. Axial T2 (A) and post-contrast T1 fat saturated (B) images in a 24 year-old male show a round hypointense and homogeneously enhancing preseptal mass abutting the globe (white arrows). No surrounding inflammatory fat stranding, swelling, or contralateral abnormality is noted. Axial diffusion-weighted imaging (DWI) (C) shows hyperintense signal compared to white matter with corresponding relatively low signal on apparent diffusion coefficient (ADC) map (D) in the mass, suggesting a cellular neoplasm and favoring lymphoma. Both radiologists agreed on neoplasm. The confirmed pathologic diagnosis of this case was mucosa-associated lymphoid tissue lymphoma.

The diagnostic performance of radiologic imaging for discriminating orbital disease categories is shown in Table 3. The balanced accuracy was 0.87–0.90 for orbital infection, 0.81–0.86 for orbital inflammation, and 0.82–0.85 for orbital neoplasm. In each result, the range indicates the difference between the two radiologists. Table 4 shows the 3x3 matrix for calculating the accuracy of radiologic diagnosis in terms of orbital disease categories. The table shows how often each radiologist accurately recognized the category of orbital disease (infection, inflammation, or neoplasm) and which alternative diagnoses accounted for the errors. The representative case of each orbital disease category is described in Figs 1–3.

**Table 3:** Diagnostic performance of radiologic imaging for discriminating 3 orbital disease categories.

	Imaging modality	Balanced accuracy	Sensitivity	Specificity	Pos pred value	Neg pred value
Radiologist 1	Orbital infection	0.90	0.86	0.95	0.57	0.99
	Orbital inflammation	0.86	0.87	0.85	0.95	0.67
	Orbital neoplasm	0.85	0.78	0.92	0.68	0.95
Radiologist 2	Orbital infection	0.87	0.79	0.96	0.61	0.98
	Orbital inflammation	0.81	0.87	0.76	0.92	0.65
	Orbital neoplasm	0.82	0.71	0.92	0.65	0.94

Pos pred value, Positive predictive value; Neg pred value, Negative predictive value.

**Table 4:** A matrix of clinical diagnosis by radiological diagnosis in terms of 3 orbital disease categories.

	Radiologic diagnosis	Clinical diagnosis (n = 191)		
		Orbital infection (n = 14)	Orbital inflammation (n = 144)	Orbital neoplasm (n = 33)
Radiologist 1	Orbital infection	12	8	1
	Orbital inflammation	1	124	6
	Orbital neoplasm	1	11	25
	Others^a^	0	1	1
Radiologist 2	Orbital infection	11	7	0
	Orbital inflammation	2	119	9
	Orbital neoplasm	1	11	22
	Others^a^	0	7	2

^a^ These scans were excluded from the matrix analysis.

Regarding the 144 scans in the orbital inflammation category, radiologic diagnosis was orbital infection in 8 scans (TED n = 2, NSOI n = 6) for radiologist 1 and 7 scans (TED n = 2, NSOI n = 2, sarcoidosis n = 2, GPA n = 1) for radiologist 2, whereas radiologic diagnosis was orbital neoplasm in 11 scans for radiologist 1 (NSOI n = 5, IgG4 n = 2, Sarcoid n = 3, ECD n = 1) and 11 scans for radiologist 2 (NSOI n = 7, IgG4 n = 1, Sarcoid n = 3). Radiologist 1 read one scan (atypical lipomatous tumor) as normal and could not interpret one scan because of a technical issue, whereas radiologist 2 read 9 scans (6 scans of TED, 1 scan of NSOI, and 2 scans of atypical lipomatous tumor) as normal.

The diagnostic performances of CT and MRI for discriminating orbital disease categories were separately analyzed. All cases of orbital cellulitis were evaluated by CT and there was no case evaluated by MRI. Diagnostic abilities discriminating orbital inflammation and orbital neoplasm categories were evaluated and are shown in Table 5. The balanced accuracies of two image modalities are comparable: The balanced accuracy of MRI was 0.85 and the balanced accuracy of CT was 0.82–0.86.

**Table 5:** Diagnostic performance of magnetic resonance image and computed tomography for discriminating orbital inflammation and orbital neoplasm.

	Imaging modality	Balanced accuracy	Sensitivity	Specificity	Pos pred value	Neg pred value
Radiologist 1	MRI	0.85	0.88	0.82	0.96	0.60
	CT	0.86	0.94	0.79	0.95	0.75
Radiologist 2	MRI	0.85	0.88	0.82	0.95	0.60
	CT	0.82	0.95	0.69	0.93	0.76

Pos pred value, Positive predictive value; Neg pred value, Negative predictive value.

**Fig 4:**
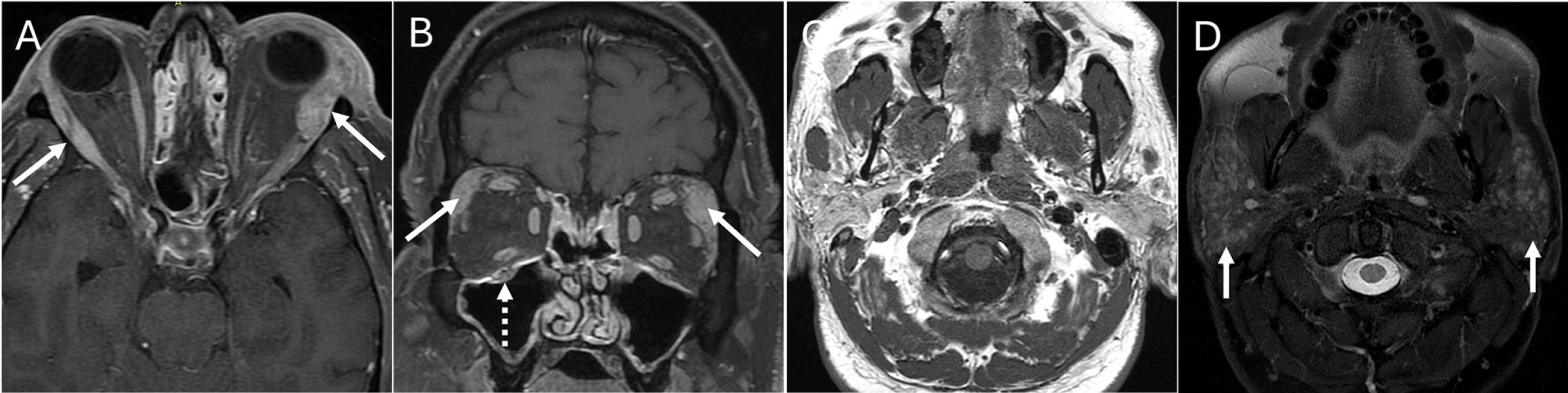
IgG4-related disease (IgG4-RD). Axial (A) and coronal (B) enhanced T1 fat saturated images in a 58 year-old male show bilateral enlarged enhancing lacrimal glands (white arrows) and right infraorbital nerve enlargement (dashed white arrow), a constellation suggesting IgG4-RD. Non-contrast axial T1 (C) and STIR (D) images show numerous small intraparotid nodules (white arrows). Both radiologists favored inflammatory disease and IgG4-RD specifically given infraorbital nerve involvement & the pattern of bilateral lacrimal & parotid involvement, consistent with the clinical diagnosis.

**Fig 5:**
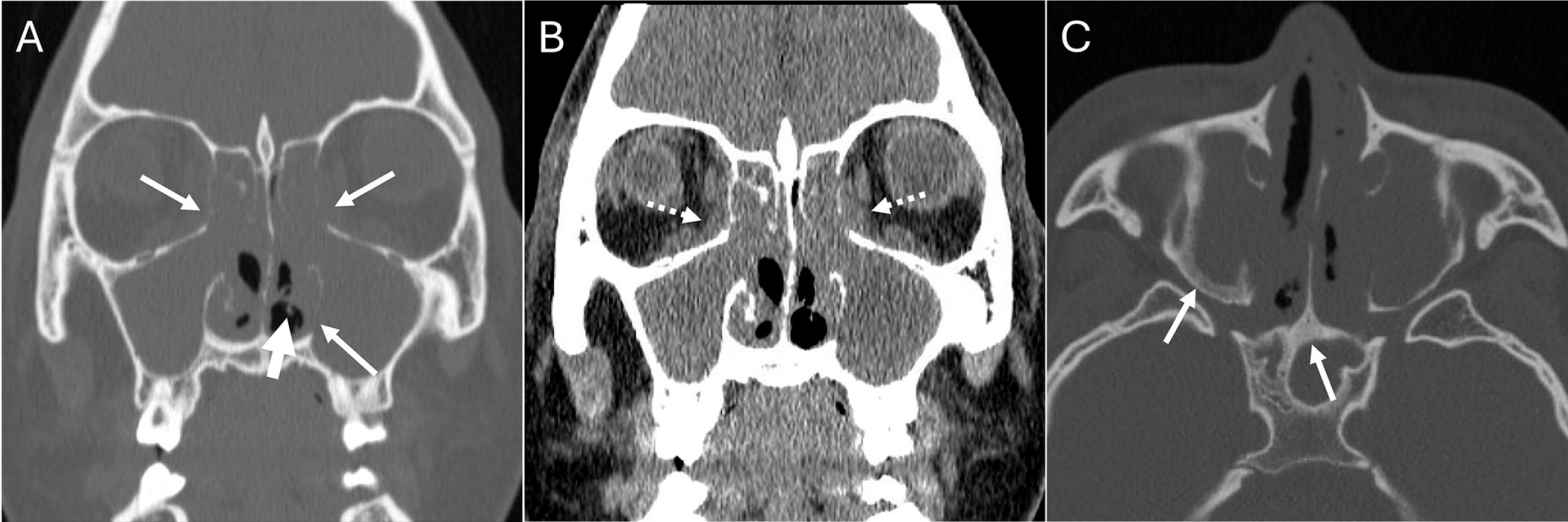
Granulomatosis with polyangiitis (GPA). Coronal bone (A) and soft tissue (B) window non-contrast CT of the sinuses in a 22 year-old male shows typical pan-sinus soft tissue opacification and nasal strands (thick arrow, A), foci of osseous erosion (thin arrows, A) and soft tissue extension into the adjacent orbits (white dashed arrows, B). Axial bone window (c) shows sclerosis of sinus walls (arrow) typical of chronic inflammatory disease and particularly GPA. Both radiologists agreed on the diagnosis of inflammation and GPA, consistent with the clinical diagnosis.

An analysis of radiologic accuracy was also performed for the condition in which the radiologist was allowed to list a second and third alternative diagnosis. If we accepted only one diagnosis, the overall accuracy was 0.69 (95% CI 0.59–0.77) for radiologist 1 and 0.74 (0.65–0.82) for radiologist 2. The balanced accuracy was highest in GPA (0.98–0.99), followed by TED (0.80–0.86), IgG4-RD (0.75–0.77), and NSOI (0.69–0.75). The balanced accuracy for Sarcoid was low (0.48–0.50). When we allowed up to 3 diagnoses, the overall accuracy improved to 0.82 (95% CI 0.74–0.88) for radiologist 1 and 0.87 (95% CI 0.80–0.03) for radiologist 2. Images of cases in the orbital inflammatory disease category were shown in Figs 4 and 5.

## Discussion

In the current study, we demonstrate several novel findings. Radiologic diagnosis is highly accurate even though the radiologists were blinded without clinical information for classifying orbital infection, orbital inflammation, and orbital neoplasm. The balanced accuracies are over 0.8 in all three orbital disease categories. However, these results do not allow the conclusion that orbital imaging should be used as the single diagnostic method for orbital diseases. Atypical infection or diffuse tumor including lymphoma and metastatic tumor are not easily differentiated from inflammation using radiologic images. In this study, there were 1 case of orbital infection and 3 cases of orbital neoplasm which were interpreted as orbital inflammation by both radiologists. CT and MRI show comparable diagnostic accuracy in terms of differentiating orbital inflammation and orbital neoplasm. Orbital inflammation subgroup analysis shows radiologic diagnostic accuracy is high accurate in GPA and TED, fair in IgG4-RD and NSOI, and disappointing in Sarcoid, although the number of cases of Sarcoid included in this series is small.

To date, there have been only a few studies describing the accuracy of radiologic diagnosis for orbital diseases. Koukkoulli et al [11] analyzed the diagnostic accuracies of clinical and radiologic diagnosis compared to the gold standard of pathologic diagnosis. They reported the accuracy of clinical diagnosis was 35.7%, whereas the radiologic diagnosis was in concordance with the pathologic diagnosis in 30.4%. In addition, they reported the sensitivity of radiologic diagnosis was 14.3% for inflammatory lesions, 28.6% for primary malignant neoplastic lesions, and 100% for secondary neoplastic lesions. Generally, the accuracy of radiologic diagnosis of this study is much lower than our results, but direct comparison between two studies is not possible. They included patients who had a pathologic diagnosis and excluded orbital infection or TED. The disease spectrum was more heterogeneous than our study. The subjects of our study were classified into orbital inflammation, orbital infection, and orbital neoplasm, while they included more diverse orbital diseases including vascular diseases and benign structural lesions.

In the present study, the positive predictive value for orbital infection or orbital neoplasm was lower than for orbital inflammation (0.57–0.61 for orbital infection, 0.65–0.68 for orbital neoplasm, and 0.92–0.95 for orbital inflammation). On the other hand, radiologic imaging showed high negative predictive values for orbital infection (0.98–0.99) and orbital neoplasm (0.94–0.95), suggesting imaging alone can be used to exclude orbital infection and neoplasm. This finding is significant in the clinic considering these two entities need urgent diagnosis and treatment. These results are affected by the lower prevalence of orbital infection (7.3%) and orbital neoplasm (17.3%) compared to orbital inflammation (75.4%) amongst the studied imaging. A more balanced image data set reflecting the actual frequency of each classification would help calculate more accurately the diagnostic abilities. The reported prevalence of orbital lesions has shown great variation depending on the materials reviewed, and classification system of the diseases. Shields et al [12] reviewed 1264 patients referred for a suspected orbital mass and reported frequencies of 11% for inflammatory lesion and 5% for TED, corresponding to 16% of orbital inflammation classification. Most of their cases were benign space-occupying orbital masses and there was no case with orbital infection. Meanwhile, Kim et al [13] reviewed 6323 patients with orbital disease in south India and reported over 50% of orbital inflammation (23.2% for idiopathic orbital inflammation and 30.6% for TED) and 4.2% of orbital infection, a similar distribution to our image set library. They used tissue origin-based classification for orbital tumors, so it was impossible to differentiate accurately benign and malignant orbital masses.

Interestingly, in the cases reviewed in our series the radiologic diagnosis was normal in a few cases with orbital diseases and most of these cases were TED. There are some typical radiologic characteristics associated with TED including exophthalmos, orbital fat expansion, extraocular muscle enlargement, and lacrimal gland enlargement [14–16]. However, in mild TED patients, only eyelid changes such as eyelid retraction, temporal flare, and eyelid lag might be present without distinct orbital changes. Therefore, clinical orbital examinations and serologic tests would help to detect these early TED patients. One case of atypical lipomatous tumor was reviewed as normal by radiologists. Atypical lipoid tumor is extremely rare in the orbit and there have been fewer than 30 cases in the literature [17,18]. In addition, the signal intensity of a fat-containing soft tissue mass can be quite similar to orbital fat, leading to difficult interpretation.

One notable finding in this study is similar diagnostic accuracy between CT and MRI for differentiating orbital inflammation and orbital neoplasm. The balanced accuracy of CT was 0.82–0.86 for CT and 0.85 for MRI. Generally, MRI is considered to have superior soft tissue contrast and spatial resolution compared to CT, so we presupposed that MRI would show higher diagnostic accuracy for differentiating orbital inflammation and neoplasm [15]. In the current study, we could not confirm the superiority of MRI over CT. We presumed that these results were influenced by the practice pattern in clinics. MRI cases may reflect a more difficult subset of disease to diagnose because MRI seems to be ordered when the disease entity is uncertain or if there is a higher suspicion for neoplasm. In addition, MRI images were taken at different institutions with variable protocols and diffusion-weighted magnetic resonance imaging (DWI) was lacking in many cases. DWI was reported to be helpful for differentiating orbital inflammation and lymphoma [19,20]. Because lymphoma is the most common malignancy in the orbit, the diagnostic accuracy of MRI would be enhanced if DWI sequences were included. A brief summary of the utility of orbital imaging in the evaluation of orbital lesions is provided in Table 6.

**Table 6:** The utility of orbital imaging in the assessment of orbital diseases.

Structure or tissue origin of the lesion and uni- vs bilaterality Size and shape of the lesion and mass effect on or involvement of adjacent structures Lesion location Lesion relationship to surrounding bone or soft tissue
CT • Internal density of the lesion / enhancement pattern • Sensitive for bone (destruction/erosion), lesion calcification, air, and paranasal sinuses
MRI • T1/T2-weighted signal intensity of the lesion / enhancement pattern • Superior soft tissue contrast, especially optic nerve and orbital apex • Cellularity of the lesion (diffusion-weighted images)

In the previous pilot study, we analyzed the accuracy rate of orbital imaging to diagnose various orbital inflammatory diseases and reported high accuracy in GPA, IgG4-RD, NSOI and TED, whereas Sarcoid showed low accuracy [10]. In the current study, we also performed the subgroup analysis based on the category of orbital inflammation and the results confirmed those of the previous study. The balanced accuracy was highest in GPA (0.98–0.99), followed by TED (0.80–0.86), IgG4-RD (0.75–0.77), and NSOI (0.69–0.75). The balanced accuracy of Sarcoid (0.48–0.50) and ECD was low (0.50), but these are also two of the rarest causes of orbital inflammation. The high balanced accuracy of orbital imaging despite blinded clinical information suggests an imaging study is useful for common orbital inflammatory diseases such as TED, NSOI, and IgG4-RD. On the contrary, it was difficult to diagnose Sarcoid by orbital imaging alone. Although sarcoidosis has characteristic pulmonary findings in chest x-ray or chest CT, there are no specific radiologic findings for diagnosing orbital Sarcoid [21,22]. Dacryoadenitis is the most common orbital manifestation of Sarcoid, but dacryoadenitis is common in other orbital inflammatory diseases.

This study has several limitations. The major limitations have been discussed above; some are inherent in the retrospective study design and the variability in disease frequencies in different geographic areas. The reference standard for the diagnostic accuracy of orbital imaging was the clinical diagnosis which was confirmed by histopathology in most cases. However, the diagnoses of orbital infection and TED were made without biopsy reflecting the typical clinical practice. This study is based on our pilot study [10]. Although some images from the smaller, more narrow pilot study were included in the present study, the previous study was conducted over 3 years ago. While we doubt that this substantially affected the current radiologic diagnosis, we cannot exclude some element of recall bias.

In conclusion, radiological images showed favorable accuracy for diagnosing various orbital disease categories. CT and MRI showed comparable abilities for differentiating orbital inflammation from orbital neoplasm. In the orbital inflammation subgroup, radiologic imaging was highly accurate for diagnosing TED, NSOI, and IgG4-RD. These findings suggest that orbital imaging is independently helpful to distinguish orbital diseases in clinical practice and can assist in differential diagnosis. Future studies are indicated to determine if other centers and other radiologists can confirm or refute our findings. Additional studies should investigate the diagnostic accuracy gained by machine learning to complement the current radiologic approach. Integration with machine learning techniques could potentially enhance the accuracy and efficiency of radiologic diagnoses. Both the patient and clinician must ultimately decide what level of uncertainty is acceptable before proceeding along a treatment plan.

## Supporting information

S1 File The minimum raw dataset analyzed in the current study. (XLSX)
